# Evaluation of Semi-Solid-State Fermentation of *Elaeocarpus serratus* L. Leaves and Black Soymilk by *Lactobacillus plantarum* on Bioactive Compounds and Antioxidant Capacity

**DOI:** 10.3390/foods10040704

**Published:** 2021-03-26

**Authors:** Chia-Yu Tsui, Chun-Yao Yang

**Affiliations:** Department of Food Science, Fu Jen Catholic University, No. 510, Zhongzheng Rd., Xinzhuang District, New Taipei City 242062, Taiwan; mimikitty28@gmail.com

**Keywords:** fermentation, *Lactobacillus plantarum*, *Elaeocarpus serratus* L., black soymilk, isoflavones, myricitrin, antioxidant capacity

## Abstract

*Elaeocarpus serratus* L. leaves (EL) containing phenolic compounds and flavonoids, including myricitrin with pharmacological properties, could be valorized as nutritional additive in foods. In this study, the semi-solid-state fermentation of EL and black soymilk (BS) by *Lactobacillus plantarum* BCRC 10357 was investigated. Without adding EL in MRS medium, the β-glucosidase activity of *L. plantarum* quickly reduced to 2.33 ± 0.15 U/mL in 36 h of fermentation; by using 3% EL, the stability period of β-glucosidase activity was prolonged as 12.94 ± 0.69 U/mL in 12 h to 13.71 ± 0.94 in 36 h, showing positive response of the bacteria encountering EL. Using *L. plantarum* to ferment BS with 3% EL, the β-glucosidase activity increased to 23.78 ± 1.34 U/mL in 24 h, and in the fermented product extract (FPE), the content of myricitrin (2297.06 μg/g-FPE) and isoflavone aglycones (daidzein and genistein, 474.47 μg/g-FPE) at 48 h of fermentation were 1.61-fold and 1.95-fold of that before fermentation (at 0 h), respectively. Total flavonoid content, myricitrin, and ferric reducing antioxidant power in FPE using BS and EL were higher than that using EL alone. This study developed the potential fermented product of black soymilk using EL as a nutritional supplement with probiotics.

## 1. Introduction

Phenolic compounds found in plant materials are known to have many biological benefits for human health. *Elaeocarpus serratus* L. is an evergreen tree widely planted in the subtropical and tropical Asia, including Taiwan [1,2]. The fruit of *E. serratus* contains carbohydrates, protein, polyphenolic compounds (flavonoids, condensed tannins, etc.), carotenoids, and vitamins [3,4]. The leaves of *E. serratus* are the by-product after harvest of fruit, and have significant quantities of polyphenols and flavonoids, including myricitrin with antioxidant activity and pharmacological properties [5,6,7,8]. Therefore, the *E. serratus* leaves are appropriate to be valorized for sustainability.

Geetha et al. (2018) reported that the ethanolic extracts of leaf and seeds from *E. serratus* had anti-arthritic activity in Wistar rats using oral administration. They also performed the acute toxicity studies of the extracts to Wistar male rats and indicated that the extracts were quite safe with no acute toxicity, even at a high dose of 5000 mg/kg b.w. orally administered [9]. Recently, Islam et al. (2020) reported that the growth performance, blood parameters, meat quality, and oxidative stability of broiler were improved by using the three-day fermentation product of *E. serratus* leaves with probiotics as the dietary supplement in feed [10]. Thus, the fermented product of *E. serratus* leaves had significant advantages as the additive in feed of broiler. For the preparation of nutraceuticals, the extracts and fermented product of *E. serratus* leaves have the potential to be used as a nutritional supplement in foods, such as in black soymilk product.

Black soybean (*Glycine max* (L.) Merr.), as the soybean with black seed coat, is rich in anthocyanin antioxidants and isoflavones, and has been considered as health food with excellent physiological functions, such as detoxification, anti-inflammatory, relieving kidney disease, and anti-aging effects [11,12]. Black soybean can be used to produce black soymilk that contains valuable isoflavones for human health. Among the isoflavones, the isoflavone glycosides are hardly absorbed by intestinal epithelium tissue and less bioactive than their aglycones [13]. Increasing the content of isoflavone aglycones would be beneficial for the consumption of black soymilk product. The isoflavone glycosides can be bio-transformed into their aglycones by the enzyme β-glucosidase, which could be released from lactic acid bacteria. Lactic acid bacteria are probiotics with benefits for the host in prevention of cancer and inhibition of intestinal pathogens and are favorable to be used for fermentation of soymilk [14,15,16].

The combination of plant foods and lactic acid bacteria fermentation was able to improve the bioactive compounds and antioxidant activity of the fermented product [17,18,19,20]. However, the compounds in plant materials with antimicrobial properties may influence the growth of lactic acid bacteria by altering the functional performance due to the response and adaptation of the bacteria to the change of environment, including culture medium and fermentation type [21,22]. *Lactobacillus plantarum* is a species of Gram-positive bacteria with flexible adaptation and high resistance to stress when encountering different environmental and plant niches [23,24,25]. Such adaptation could be useful to be applied in the fermentation of plant materials. In addition, *L. plantarum* has been validated as functional probiotics for industrial fermentation of traditional Italian sausages [26].

By combining the functionalities of *E. serratus* leaves (EL), black soymilk (BS), and *L. plantarum*, the bioactive compounds in the fermented product might be enriched for health benefits. Thus, the aim of this study was to investigate the effect of semi-solid-state fermentation of EL and BS by using *L. plantarum* BCRC 10357 on bioactive compounds and antioxidant capacity of the fermented product. The growth behavior of *L. plantarum* BCRC 10357 encountering the presence of EL in the culture medium was explored to evaluate the response of the bacteria in β-glucosidase activity. With the most appropriate amount of EL in BS medium for the fermentation by *L. plantarum* BCRC 10357, the growth of the bacteria, total flavonoid content, antioxidant capacity, myricitrin content, isoflavone glycosides (daidzin and genistin), and their aglycones (daidzein and genistein) in the fermented product were analyzed to evaluate the efficiency of fermentation. Furthermore, the surface morphology and functional groups in the solid parts separated from the fermentation medium were also analyzed by field emission scanning electron microscope (FE-SEM) and Fourier transform infrared spectroscopy (FTIR), respectively, to verify the effect of fermentation on the surface structures of *E. serratus* leaves with *L. plantarum*. This study developed the potential fermented product of black soymilk using *E. serratus* leaves as a nutritional supplement with *L. plantarum*.

## 2. Materials and Methods

### 2.1. Materials

The *Elaeocarpus serratus* L. leaves were collected in January 2019 from the orchard (23°29′18.1″ N 120°28′15.7″ E) in Chiayi City, Taiwan, and were thoroughly rinsed with distilled water to remove the dust on the surface [5]. After being freeze-dried, the leaves were crushed, ground, and screened with 60-mesh sieves to obtain a powder of *E. serratus* L. leaves (EL), which was stored at 4 °C for use.

The black soymilk used in the fermentation was prepared from Tainan No. 3 black soybean (*G. max* (L.) Merr., place of origin in Tainan), which was harvested at the end of 2019 and purchased from Shia Ying Farmers’ Associations in Tainan County, Taiwan. The preparation of black soymilk followed the procedure of Peng et al. (2018) with a slight modification [24]. The black soybean was thoroughly cleaned by rinsing and soaked in the ratio 1:10 (*w*/*v*) of soybean to distilled water for overnight. After the draining of water, the black soybean and distilled water in the ratio of 1:6 (*w*/*v*) were homogenized for 3 min, and the mixture was then filtered to get the black soymilk, which was stored at 4 °C for use.

The reagents, (±)-6-hydroxy-2,5,7,8-tetramethylchromane-2-carboxylic acid (Trolox), *p*-nitrophenol β-D-glucopyranoside (*p*-NPG), 2,4,6-tris(2-pyridyl)-s-triazine (TPTZ), myricitrin, rutin, daidzein, daidzin, genistein, and genistin used as standards were purchased from Sigma-Aldrich (St. Louis, MO, USA). Other reagents were purchased from Won-Won Biotechnology Co., Ltd. (New Taipei City, Taiwan), Sigma-Aldrich (St. Louis, MO, USA), and Merck (Darmstadt, Germany).

The experimental steps, methods, and analysis are described below, and are summarized in a scheme as Figure S1 in Supplementary Materials.

### 2.2. Lactobacillus plantarum BCRC 10357 Culture and Growth

*Lactobacillus plantarum* BCRC 10357 was obtained from Bioresource Collection and Research Center, Food Industry Research and Development Institute (Hsinchu, Taiwan), and the bacterial culture was preserved at −80 °C. For activation of *L. plantarum* BCRC 10357, the culture was inoculated in the sterilized de Man, Rogosa and Sharpe (MRS) broth at 37 °C for 24 h twice, and then incubated in MRS agar at 37 °C for 48 h. One colony was selected from MRS agar to incubate in MRS broth for the subsequent experiments.

The growth of *L. plantarum* was determined by using the pour plate method of the fermented broth. The fermented broth was diluted with 0.85% (*w*/*v*) sterilized saline by using 10-fold serial dilution method, and 1 mL of dilution fermented broth was taken and incubated in MRS agar at 37 °C for 48–72 h by pour plate method for the determination of viable cell counts (expressed as log CFU/mL).

### 2.3. Semi-Solid-State Fermentation of Elaeocarpus serratus L. Leaves and Black Soymilk

The fermentation medium prepared by adding x% solid EL into MRS broth (*w*/*v*) was denoted as x% EL (x = 0.5, 1, 3, or 5). The x% EL medium was sterilized at 121 °C for 20 min, and *L. plantarum* was inoculated in 1% (*v*/*v*) into the sterilized x% EL medium to start the fermentation at 37 °C for 12–48 h. The control group was the fermentation using MRS medium without adding EL, so as to evaluate the response of *L. plantarum* when encountering the plant-based material EL at different amounts.

After evaluating the response of *L. plantarum* with various %EL in MRS medium, the appropriate ratio of 3% EL was selected in the subsequent experiments. The fermentation medium prepared by adding 3% solid EL into liquid BS (*w*/*v*) was denoted as BSEL. Prior to the sterilization, the BSEL medium was sonicated at 60 °C for 3 h using ultrasonic bath equipped with frequency/power at 40 kHz/300 W (LEO-3002S, LEO Ultrasonic Co., Taiwan) for well-mixing and facilitating transport of liquid BS into internal network of solid EL. After that, the BSEL medium was sterilized at 121 °C for 20 min, and *L. plantarum* was inoculated in 1% (*v*/*v*) into the sterilized BSEL medium to start the fermentation at 37 °C for 48 h.

At the selected fermentation time, the fermented broth was separated from the medium by using centrifugation (3800 rpm at 4 °C for 10 min) and freeze-dried to get the fermented product (denoted as FP). Then, the fermented product was extracted by using 95% ethanol at 30 °C under ultrasound (40 kHz) for 4 h, and centrifuged using 4000 rpm for 10 min to get the supernatant, which was freeze-dried to obtain the fermented product extract (denoted as FPE) for subsequent analysis.

### 2.4. Determination of β-Glucosidase Activity

The measurement of the amount of *p*-nitrophenol (*p*-NP) released from the rate of hydrolysis of *p*-nitrophenyl β-D-glucopyranoside (*p*-NPG) was used to determine the β-glucosidase activity. One unit (U) of the β-glucosidase activity was defined as the quantity of β-glucosidase that released one nmol of *p*-NP from *p*-NPG per ml per min at 37 °C under assay condition [27]. The method of Liu et al. (2018) to determine β-glucosidase activity was followed with a slight modification [16]. Two hundred microliters of 5-mM *p*-NPG (prepared with 0.1M of phosphate buffer solution at pH 7.0) were introduced into 2 mL of fermentation medium (cases: x% EL in MRS, and 3% EL in BS) to react at 37 °C for 30 min. One hundred microliters of 1.0-M Na_2_CO_3_ were added to stop the reaction at 4 °C. The mixture was then centrifuged at 3800 rpm for 30 min to get the supernatant, which was filtered by 0.45-μm membrane and analyzed for β-glucosidase activity using spectrophotometer at 405 nm (Hitachi, Ratio Beam Spectrophotometer U-5100, Tokyo, Japan).

### 2.5. Determination of Total Flavonoid Content

The total flavonoid content (TFC) in the fermented product extract (FPE) was determined using rutin equivalent (RE) and followed the method of Khemakhem et al. (2017) with a slight modification [28]. The dried FPE was re-dissolved in 75% aqueous ethanol to prepare the liquid sample of extract for analysis. One hundred and twenty five microliters of the liquid sample of extract were mixed with 75 µL of 5% NaNO_2_ and 500 μL of de-ionized water to react at room temperature for 5 min. Then, 37.5 µL of 10% AlCl_3_ and 500 μL of NaOH (1 M) were added in turn into the mixture. The absorbance of the mixture was measured using a spectrophotometer at 510 nm to determine TFC, which was expressed as µg-RE/g-FPE or µg-RE/g-FP.

### 2.6. Determination of Ferric Reducing Antioxidant Power

The method of Shirzad et al. (2017) was followed with some modifications to determine the antioxidant activity of the fermented product extract by ferric reducing antioxidant power (FRAP) using trolox equivalent (TRE) [29]. The liquid sample of extract was prepared as described in Section 2.5. The FRAP reagent was prepared and preserved at 37 °C. The mixture of 285-μL FRAP reagent, 15-μL liquid sample of extract, and 500-μL deionized water was reacted at 37 °C for 4 min. The absorbance of the mixture was measured using spectrophotometer at 593 nm to determine the FRAP, which was expressed as mg-TRE/g-FPE or mg-TRE/g-FP.

### 2.7. HPLC Analysis of Myricitrin and Isoflavones

The content of myricitrin and isoflavones in the fermented product extract were determined by using high performance liquid chromatography (HPLC) with standards of myricitrin, daidzein, daidzin, genistein, and genistin (Sigma-Aldrich, USA) for calibration and quantification. The HPLC system was equipped with diode Array-detector (JASCO, MD-2010, Tokyo, Japan) and Mightysil RP-18 GP column (5 μm, 250 mm × 4.6 mm, Kanto Chemical Co., Tokyo, Japan). The HPLC conditions followed the method of Chen and Yang (2020) for myricitrin analysis [5] and the method of Yu and Yang (2019) for isoflavones analysis [30]. With the solvent A (acetonitrile) and solvent B (0.1% trifluoroacetic acid) as the mobile phase, the gradients were set as: (1) for myricitrin analysis, solvent A: 10 to 20% at 0 to 10 min, 20 to 40% at 10 to 35 min, 40 to 100% at 35 to 40 min, 100% at 40 to 45 min, and 100 to 10% at 45 to 46 min, (2) for isoflavones analysis, solvent A: 10% at 0 to 10 min, 10 to 55% at 10 to 35 min, 55 to 10% at 35 to 45 min, and 10% at 45 to 60 min.

### 2.8. FE-SEM and FTIR Analysis

The solid parts after separating the fermented broth from the medium were analyzed for surface morphology by field emission scanning electron microscope (FE-SEM) (JEOL, JSM-7800F, Tokyo, Japan) and functional groups by Fourier transform infrared spectroscopy (FTIR) (FT-730, Horiba, Kyoto, Japan) within the range of wave numbers 400–4000 cm^−1^ to verify the effect of fermentation on the structural changes of EL, and were denoted as S-3%EL-time for 3% EL medium and S-BSEL-time for BSEL medium at selected fermentation time.

### 2.9. Statistical Analysis

Triplicate experiment for each condition was performed with three independent samples, and the experimental data were expressed as mean ± standard deviation (*n* = 3). Statistical analysis was evaluated by one-way analysis of variance (ANOVA) with Duncan’s multiple range test using IBM SPSS Statistics 20 (IBM SPSS Statistics for Windows v. 20.0, IBM Corp, Armonk, NY, USA). The significant difference was determined at *p* < 0.05.

## 3. Results and Discussion

### 3.1. Response of L. plantarum BCRC 10357 Incubated by Elaeocarpus serratus L. Leaves in MRS

**Figure 1:**
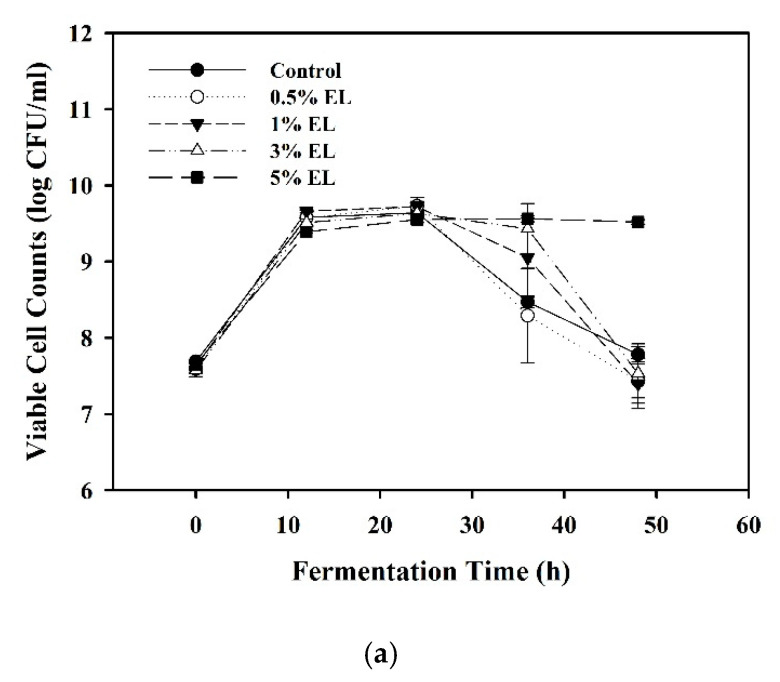

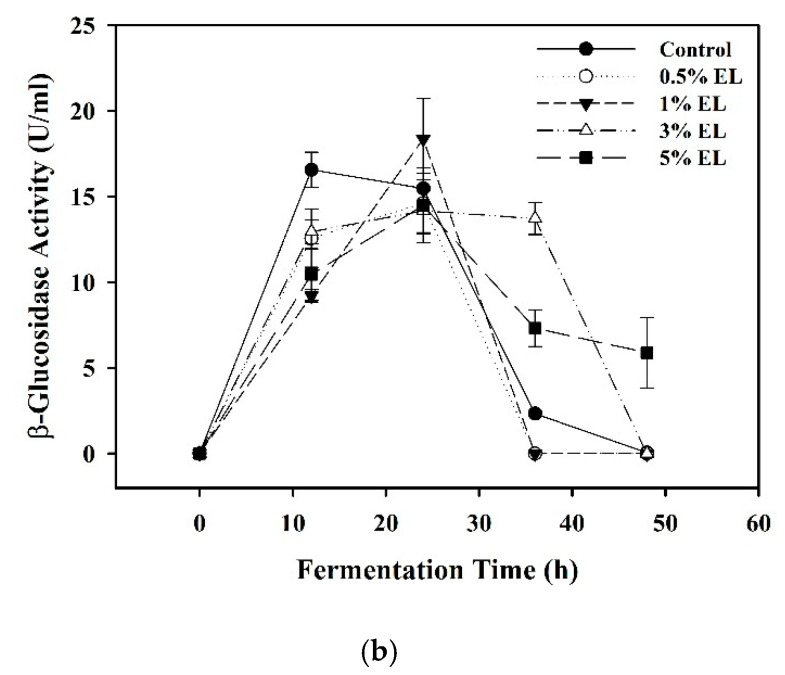
Effect of various percentages of *E. serratus* leaves (EL) in MRS on the growth of *L. plantarum*: (**a**) viable cell counts vs. fermentation time; (**b**) β-glucosidase activity vs. fermentation time. The data were expressed as mean ± standard deviations (*n* = 3).

For the fermentation of EL by *L. plantarum* BCRC 10357, the response of the bacteria encountering the solid EL in the MRS broth was explored on their growth behavior and β-glucosidase released. Various percentages (0.5, 1.0, 3.0, and 5.0%) of EL were added in MRS medium to evaluate the adaptive evolution of *L. plantarum* to different amounts of EL in the medium. The MRS broth without adding EL was used as the control. The viable cell counts and β-glucosidase activity against fermentation time are shown in Figure 1a,b, respectively. The tests for significant differences are displayed in Tables S1 and S2 in Supplementary Materials.

As shown in Figure 1a, before 24 h of fermentation, the viable cell counts using various amounts of EL in the MRS medium did not vary much with that of the control. However, at 36 h of fermentation, the order of viable cell counts (log CFU/mL) was 5% EL (9.56 ± 0.04) > 3% EL (9.43 ± 0.33) > 1% EL (9.05 ± 0.52) > the control (8.47 ± 0.08) > 0.5% EL (8.29 ± 0.62), and the viable cell counts for the control and 0.5% EL were all greatly less than that for 3% EL and 5% EL (Table S1), showing that the stationary growth phase of *L. plantarum* was able to be prolonged by the utilization of EL.

As shown in Figure 1b, it was found that the stability of β-glucosidase activity of *L. plantarum* would be enhanced from 24 h to 36 h by the presence of 3% and 5% of EL in the MRS medium, compared to that of the control. Without adding EL in MRS medium, the β-glucosidase activity of *L. plantarum* quickly reduced to 2.33 ± 0.15 U/mL in 36 h of fermentation; while by using 3% EL, the stability period of β-glucosidase activity was prolonged as 12.94 ± 0.69 U/mL in 12 h to 13.71 ± 0.94 in 36 h without significant differences (Table S2). The best adaptive response of *L. plantarum* occurred in the condition of MRS broth with 3% of solid EL, and showed that the bioactive compounds provided by the plant material EL could be utilized by *L. plantarum* to regulate the physiological state to adapt to the particular environment encountered for survival [25], such that *L. plantarum* BCRC 10357 could release the β-glucosidase for a longer period, compared to that without using EL in the MRS medium. The results were supported by the study of Filannino et al. (2016), who investigated the plant niche-specific traits of *L. plantarum* through whole-transcriptome and phenotypic microarray profiles with carrot and pineapple juices as model systems and MRS broth as a control [25]. They concluded that the behavior and, in turn, environmental adaptation of *L. plantarum* were affected by the carrot substrate, and the strain sensed the plant stimulus to adjust its carbohydrate metabolism for increasing the capacity to compete [25]. In this study, the results demonstrated that *L. plantarum* has the ability to adapt to ferment plant substrates EL by using available nutrients for growth.

### 3.2. Effect of Elaeocarpus serratus L. Leaves on Bioactive Compounds in Fermented Product

**Figure 2:**
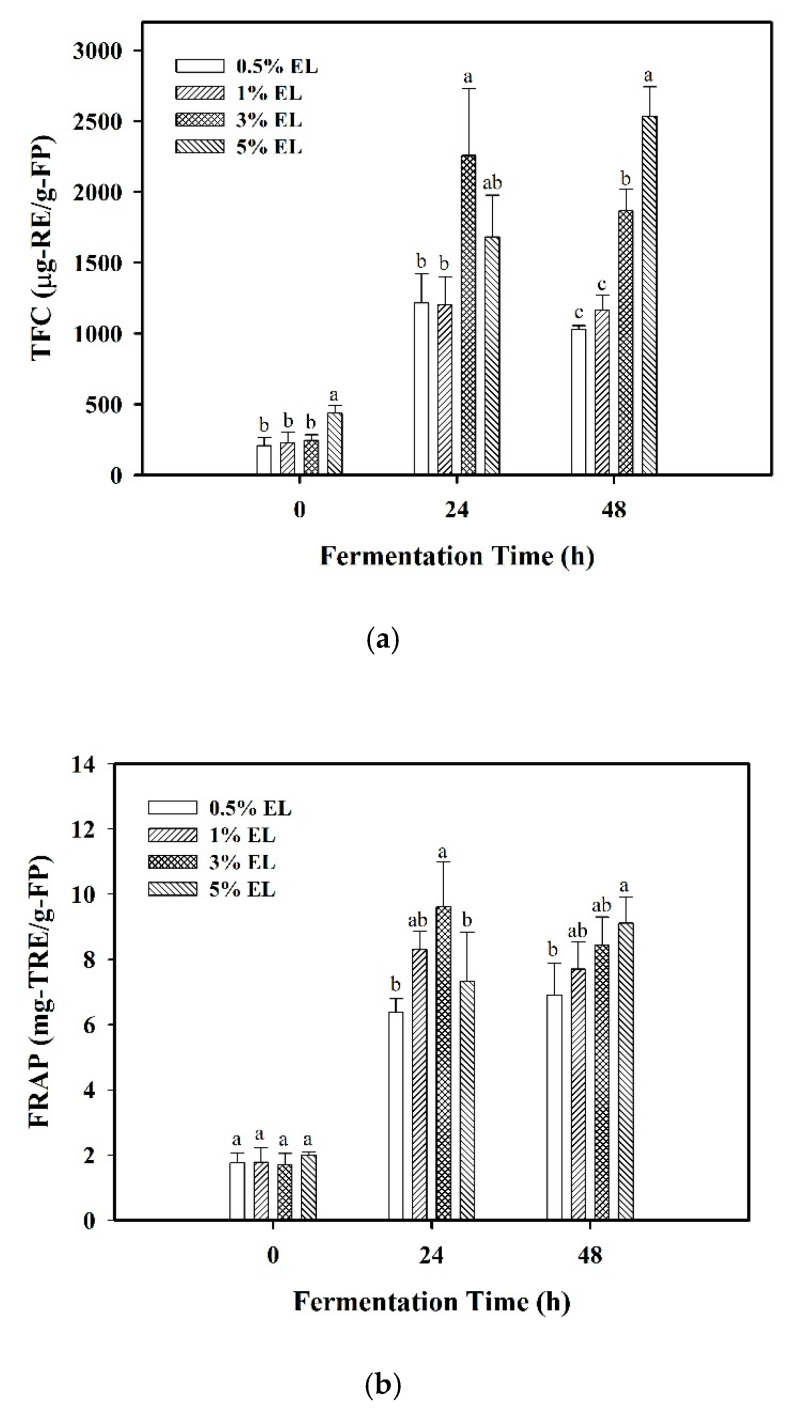
Comparison of fermented products with various %EL in MRS by *L. plantarum*: (**a**) total flavonoid content (TFC) vs. fermentation time; (**b**) ferric reducing antioxidant power (FRAP) vs. fermentation time. The data were expressed as mean ± standard deviations (*n* = 3). Different letters at the same time show significant differences among the different %EL (*p* < 0.05) by Duncan’s multiple range test.

During the fermentation of EL in MRS broth by *L. plantarum*, the bioactive compounds with hydrophilic property would be simultaneously extracted by the liquid MRS broth, and the bacteria can access the compounds available in the interior of EL for metabolism. Hence, the flavonoid compounds in the fermented broth were expected to result from the extraction and fermentation of EL. The TFC and FRAP of the fermented product by using different percentages of EL in the MRS medium are shown in Figure 2a,b, respectively. In addition, the tests for significant differences of the same %EL at various fermentation times are shown in Table S3 in Supplementary Materials. By using 3% EL in the medium, the TFC increased from 246.39 ± 38.11 μg-RE/g-FP at 0 h to 2257.94 ± 472.77 μg-RE/g-FP at 24 h of fermentation, and FRAP increased from 1.69 ± 0.36 mg-TRE/g-FP at 0 h to 9.61 ± 1.37 mg-TRE/g-FP at 24 h of fermentation. Both the TFC and FRAP of the fermented product by using 3% EL at 24 h of fermentation were the largest, compared to that of the other tested media.

However, at 48 h of fermentation, both the TFC and FRAP values all increased with the increasing amount of EL added. By using 5% of EL, the TFC increased from 437.38 ± 54.87 μg-RE/g-FP at 0 h to 1682.26 ± 296.09 μg-RE/g-FP at 24 h and further to 2533.41 ± 208.85 μg-RE/g-FP at 48 h of fermentation. The increase for TFC from 0 to 24 h was higher than that from 24 to 48 h (Table S3). The trend for the increase of FRAP with respect to time was similar to that for TFC. Such findings were consistent with the time-dependent tendency of growth behavior and β-glucosidase activity of *L. plantarum*. At fermentation time greater than 24 h, the amount of the extracted flavonoid compounds utilized by *L. plantarum* by using 3% EL was more than that by using 5% EL. Hence, the stationary growth phase of the bacteria was prolonged for survival with the β-glucosidase activity stabilized until 36 h by using 3% EL in the MRS broth. Moreover, the TFC slightly decreased from 24 to 48 h of fermentation by using 3% EL. This implied that the steric hindrance for *L. plantarum* to access and utilize EL by using 5% of solid EL in the MRS broth would be greater than that by using 3% EL.

**Figure 3:**
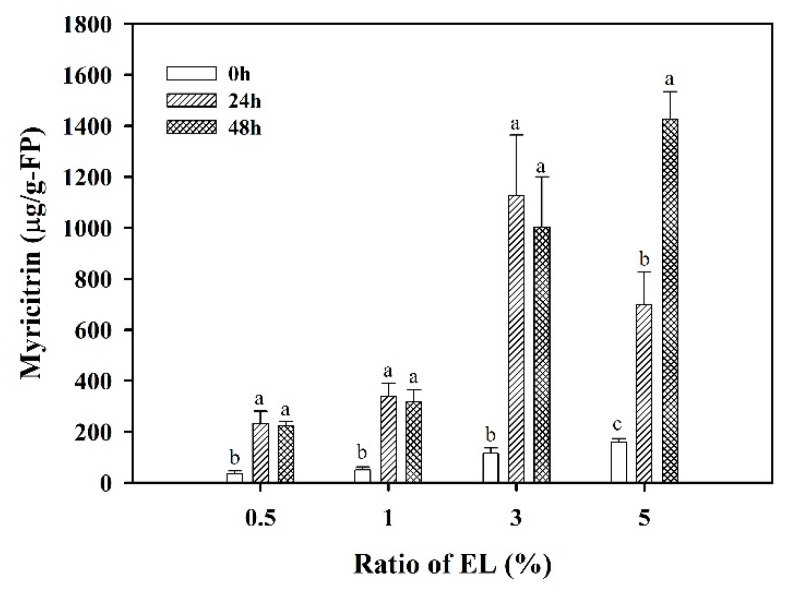
Comparison of various %EL on myricitrin content in fermented products at 0, 24, and 48 h of fermentation time. The data were expressed as mean ± standard deviations (*n* = 3). Different letters at the same %EL were significantly different (*p* < 0.05) by Duncan’s multiple range test.

Besides, the variation of the specific flavonoid compound myricitrin in the fermented product for various %EL in the MRS broth is displayed in Figure 3 with the statistical analysis for the same %EL at various times. The tests for significant differences of various %EL at the same time are shown in Table S4 in Supplementary Materials. It was found that, for 0.5%, 1%, and 3% of EL addition, myricitrin content was increased with significant difference in the comparison between 0 and 24 h of fermentation, but with insignificant difference in the comparison between 24 and 48 h, except the use of 5% EL in the medium, with which the content of myricitrin increased by increasing the fermentation time with significant difference from 0 to 48 h of fermentation. On using 3% EL, the content of myricitrin in the fermented product was 115.03 ± 20.79 μg/g-FP at 0 h, 1126.70 ± 237.46 μg/g-FP at 24 h, and 1002.41 ± 197.68 μg/g-FP at 48 h. It exhibited that, using 3% EL, the content of myricitrin at 24 h was slightly higher than that at 48 h, but with insignificant difference. Furthermore, at 24 h of fermentation, the content of myricitrin using 3% EL was higher than that using 5% EL (697.96 ± 127.82 μg/g-FP) (Table S4). The trends of myricitrin content against fermentation time using 3% EL and the comparison with 5% EL in the MRS broth were all consistent with that of the TFC and FRAP.

The trends in TFC, myricitrin content, and FRAP against fermentation time were found to correspond to the expressions of viable cell counts and β-glucosidase activity of *L. plantarum* during the 48-h fermentation. These results demonstrated that an appropriate amount of plant material, 3% EL, in the MRS broth was able to enhance the survival of *L. plantarum* with the β-glucosidase activity stabilized at a high level in a prolonged period. In addition, the fermented product from the extraction and fermentation of *E. serratus* leaves contained bioactive compounds with antioxidant activity and could be utilized as the potential nutrient source.

### 3.3. Response of L. plantarum Incubated by Black Soymilk and E. serratus Leaves

From the investigation of EL fermented by *L. plantarum*, the optimal addition of 3% EL in the MRS medium was selected for application in the fermentation of black soymilk. The black soymilk contains isoflavone glycosides (daidzin and genistin) and a relatively small amount of isoflavone aglycones (daidzein and genistein), and the isoflavone glycosides can be bio-transformed into isoflavone aglycones by fermentation using β-glucosidase released by the probiotics to enhance bioavailability for human health. The combination of the functionalities of fermented black soymilk and EL was explored. The fermentation of soymilk with 3% of EL was conducted in the semi-solid-state fermentation manner by *L. plantarum* BCRC 10357 at 37 °C for fermentation time of 0 to 48 h. The adaptive response of *L. plantarum* BCRC 10357 in viable cell counts and β-glucosidase activity by using the fermentation media of BSEL and 3% EL in MRS broth at 0, 24, and 48 h is shown in Table 1.

The viable cell counts using BSEL at 48 h was 8.02 ± 0.54 log CFU/mL, which was greater than that using 3% EL, but with insignificant difference. At the start of fermentation (at 0 h), there were no β-glucosidase activity observed for both media 3% EL and BSEL. At 24 h of fermentation time, the β-glucosidase activity using BSEL was enhanced to 23.78 ± 1.34 U/mL, which was much greater than that of 3% EL, although there were similar viable cell counts for both medium. This displayed that *L. plantarum* further regulated their physiological state to adapt to the new environment containing black soymilk and solid EL and was more active to release more β-glucosidase than that just using 3% EL in MRS broth. At 48 h of fermentation time, there was still 3.58 ± 1.30 U/mL of β-glucosidase activity released, showing the fermentation medium of black soymilk with EL facilitating *L. plantarum* to raise the positive adaptation to plant materials.

### 3.4. Semi-Solid-State Fermentation of Black Soymilk and E. serratus Leaves

The black soymilk with EL fermented by *L. plantarum* BCRC 10357 was a type of semi-solid-state fermentation. In such a system, black soymilk would transport into the interior of EL with some of the isoflavones and bioactive compounds that originally existed in the soymilk. Tew et al. (1996) studied the effect of dietary fiber on the bioavailability of isoflavones and indicated that insoluble fibers from wheat bran cereal affected the bioavailability of isoflavones, probably by the ability to bind to isoflavones [31]. The previous study of Chen and Yang (2019) reported that *E. serratus* leaves contained carbohydrates, including cellulose for converting to reducing sugars by enzymatic hydrolysis [32]. Therefore, some isoflavones and bioactive compounds in the black soymilk could be adsorbed in EL by binding to the insoluble fibers of EL during the sterilization process. The content of glycosides (daidzin and genistin) and aglycones (daidzein and genistein) isoflavones in the fermented product extract (FPE) at 0, 24, and 48 h of fermentation is displayed in Table 2.

As shown in Table 2, the content of daidzin and genistin in the fermented product extract increased from 1033.01 ± 160.26 and 853.17 ± 250.65 μg/g-FPE at 0 h (after sterilization and inoculation of bacteria) to 1372.80 ± 271.20 and 1487.71 ± 290.96 μg/g-FPE at 24 h of fermentation, and then decreased to 763.57 ± 181.47 and 1115.25 ± 224.14 μg/g-FPE at 48 h of fermentation, respectively. As mentioned above, the sterilization process of black soymilk with EL, which contained carbohydrates including cellulose [32], would make the isoflavones in black soymilk simultaneously transport into the internal EL. When the fermentation was started after inoculation of the bacteria at 0 h of fermentation, the compounds that had been adsorbed on internal EL would be extracted into the fermented product. As a result, at 0 to 24 h of fermentation time, daidzin and genistin was gradually released from the binding sites of the internal fiber or cellulose of EL into the fermented product during fermentation. When the fermentation proceeded from 24 to 48 h, the highest β-glucosidase activity (23.78 ± 1.34 U/mL at 24 h) (Table 1) released by *L. plantarum* tended to bio-transform daidzin and genistin individually into aglycone isoflavones of daidzein and genistein. The biotransformation resulted in the content of daidzin and genistin decreasing from 24 to 48 h of fermentation.

For the aglycones, the content of daidzein and genistein in the fermented product extract was increased from 187.78 ± 14.16 and 54.95 ± 22.99 μg/g-FPE at 0 h (after sterilization and inoculation of bacteria) to 283.82 ± 49.28 and 89.55 ± 22.21 μg/g-FPE at 24 h, and further increased to 339.89 ± 50.17 and 134.58 ± 42.20 μg/g-FPE at 48 h of fermentation, respectively. Due to the biotransformation of daidzin and genistin, the content of aglycone isoflavones was increased to 474.47 μg/g-FPE at 48 h, and was 1.95 folds that at 0 h. Besides, the ratio of aglycone isoflavones in the total isoflavones (daidzin, genistin, daidzein, and genistein) increased from 11.4% to 20.16% in the fermented product extract at 48 h of fermentation, due to the cellulose-containing EL having the ability to bind the β-glucosidase in the internal network to limit the catalytic function of β-glucosidase by steric hindrance [33].

**Figure 4:**
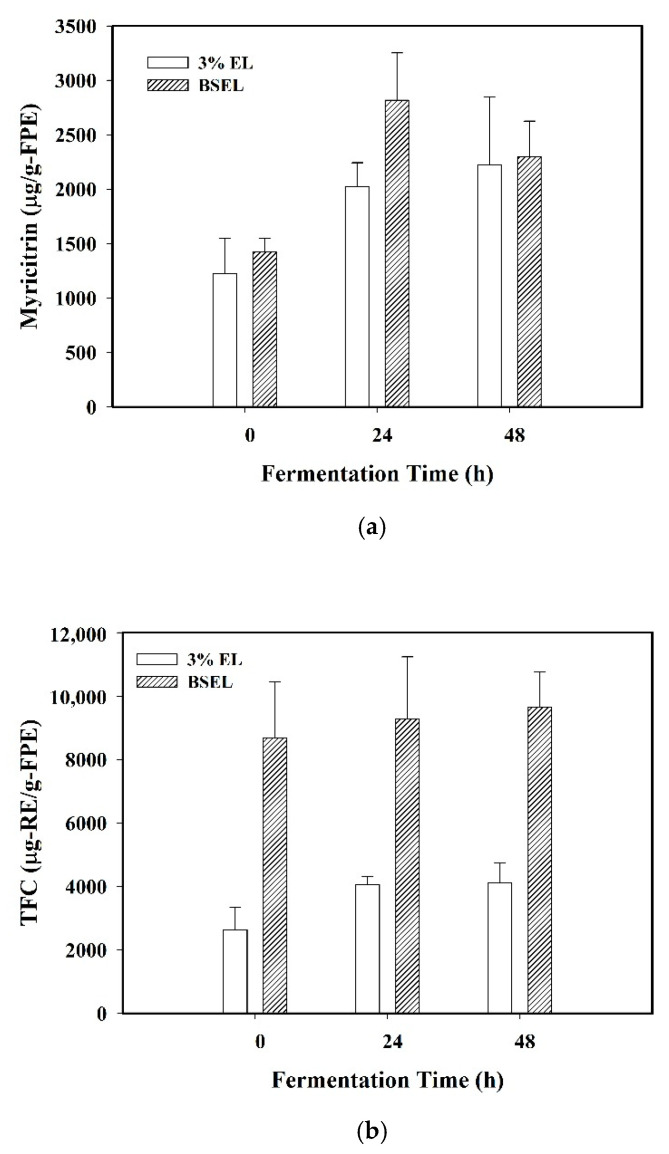

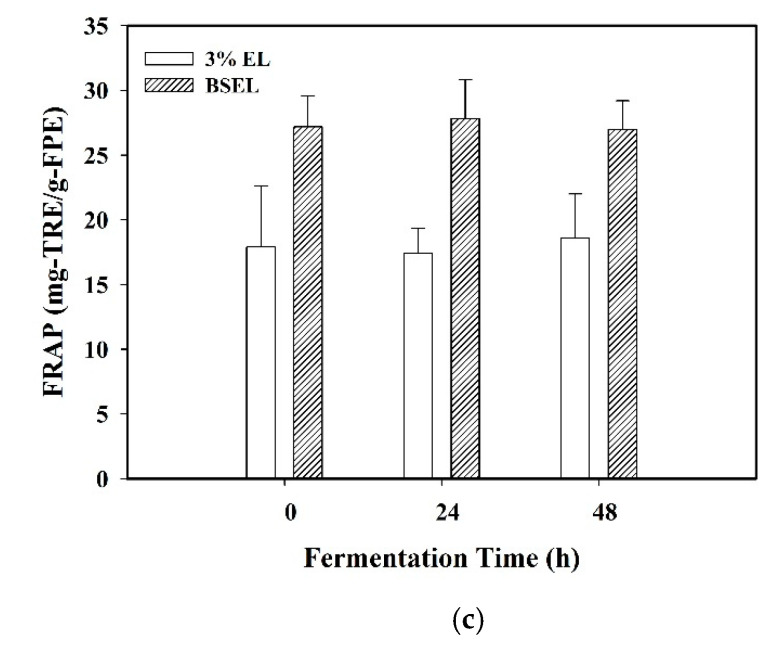
Comparison of (**a**) myricitrin content, (**b**) total flavonoid content (TFC), and (**c**) ferric reducing antioxidant power (FRAP) of the fermented product extract (FPE) at 0, 24, and 48 h of fermentation for the medium 3% EL in MRS (3% EL) and the medium 3% EL in black soymilk (BSEL). The data were expressed as mean ± standard deviations (*n* = 3).

Furthermore, the myricitrin content, total flavonoids content (TFC), and antioxidant capacity as ferric reducing antioxidant power (FRAP) of the fermented product extract using BSEL and 3% EL at 0, 24, and 48 h of fermentation are shown in Figure 4a–c. The tests for significant differences of the same medium at various times are shown in Table S5 in Supplementary Materials.

By using BSEL as the medium, the content of myricitrin in the fermented product extract was 2297.06 ± 328.35 μg/g-FPE at 48 h, which was 1.61 fold that (1426.61 ± 119.96 μg/g-FPE) at 0 h with a significant increase. The myricitrin content (2817.17 ± 435.51 μg/g-FPE) at 24 h was higher than that at 48 h, but with insignificant difference (Table S5), showing that the 24-h fermentation time was sufficient for the extraction of myricitrin from EL by the black soymilk. Using BSEL, the TFC in the fermented product extract was slightly increased with increasing fermentation time, but with insignificant difference for 9643.64 ± 1120.21 μg/g-FPE at 48 h. By using BSEL, the FRAP for the fermented product extract at 48 h was 26.98 ± 2.21 mg/g-FPE, which was insignificantly different with that at 0 and 24 h of fermentation. Undoubtedly, the FRAP values were corresponding to the TFC trend using BSEL, demonstrating that most flavonoid compounds were transported into the fermented product at 0 h of fermentation.

Moreover, the FRAP and content of myricitrin and TFC in the fermented product extract at 0, 24, and 48 h by using BSEL were all greatly higher than that by using 3% EL in MRS broth. At 24 h of fermentation, the content of myricitrin and TFC, and antioxidant capacity (FRAP) with 3% EL in black soymilk were 1.39-fold, 2.29-fold, and 1.60-fold of that with 3% EL in MRS broth, respectively. This showed that, compared with MRS broth, black soymilk was able to facilitate the transport of myricitrin and other flavonoids from EL into the fermented product during the fermentation process.

### 3.5. Surface Morphology of Solid Substrate with L. plantarum

**Figure 5:**
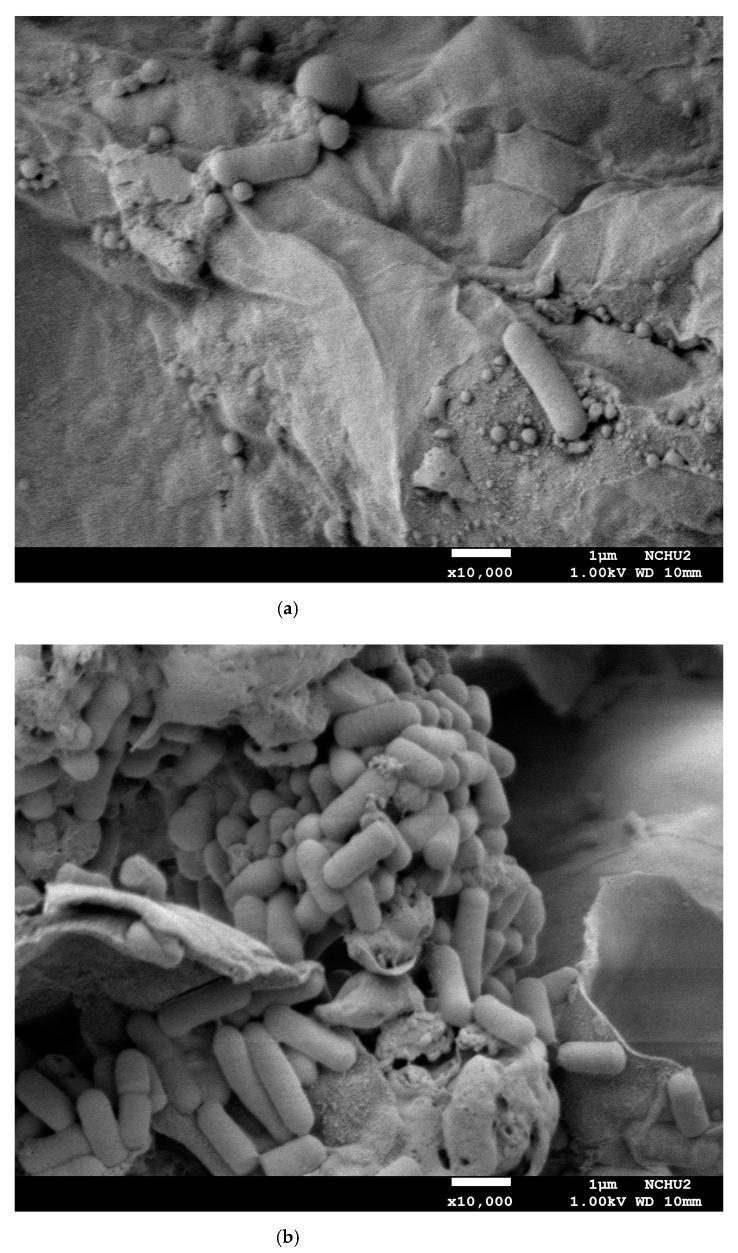

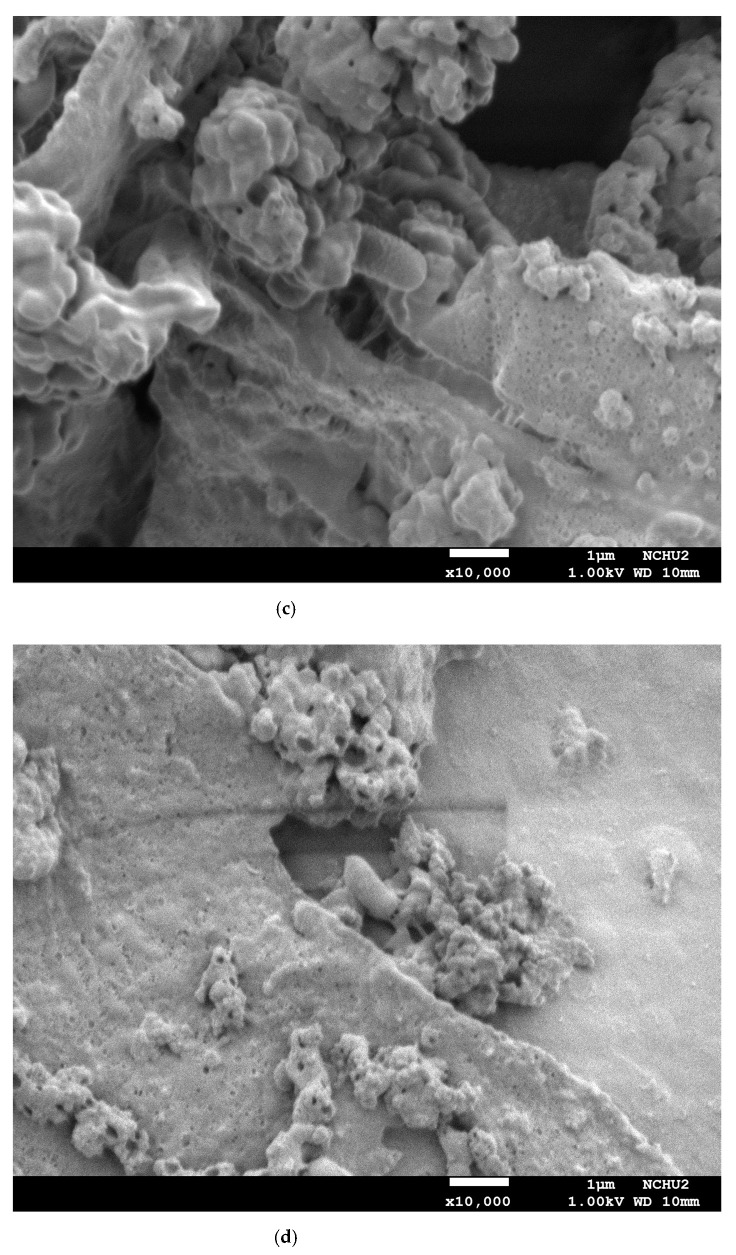
FE-SEM images of the solids separated from the sterilized medium after inoculation of *L. plantarum*: (**a**) solids of 3% EL in MRS at 0 h (S-3%EL-0 h), (**b**) solids of 3% EL in MRS at 48 h of fermentation (S-3%EL-48 h), (**c**) solids of 3% EL in black soymilk at 0 h (S-BSEL-0 h), and (**d**) solids of 3% EL in black soymilk at 48 h of fermentation (S-BSEL-48 h).

Field emission scanning electron microscope (FE-SEM) was used to observe the colony morphology of the *L. plantarum* on the surface of *E. serratus* leaves using MRS or black soymilk medium before and after fermentation. The surface morphology of the solids separated from the sterilized medium after inoculation of *L. plantarum* was observed by using FE-SEM for 0 and 48 h of fermentation time, as shown in Figure 5a–d.

As displayed in Figure 5a for solids of 3% EL in MRS at 0 h (S-3%EL-0 h), the bacteria were observed to loosely reside in a small amount on the surface of the solid EL after inoculation. In 48 h of incubation, the bacteria were increased and largely gathered to reside on the surface of solid EL, and the surface of EL was ruptured to expose the pores for diffusion of the liquid materials, as shown in Figure 5b for solids of 3% EL in MRS at 48 h of fermentation (S-3%EL-48 h).

For the incubation using black soymilk with 3% EL, after sterilization and inoculation of *L. plantarum* at the start of fermentation (at 0 h), the surface of solid EL was a little broken to present the pores for transport of components, and a small amount of bacteria adhered to reside on the surface of EL. In 48 h of incubation, the surface of EL was rough by the coverage of black soymilk with some denatured-protein particles [5], and the bacteria gathered to adhere with some ruptured denatured-protein particles on the surface of EL for the biotransformation reaction.

### 3.6. Functional Groups of Solid Substrate during Fermentation

**Figure 6:**
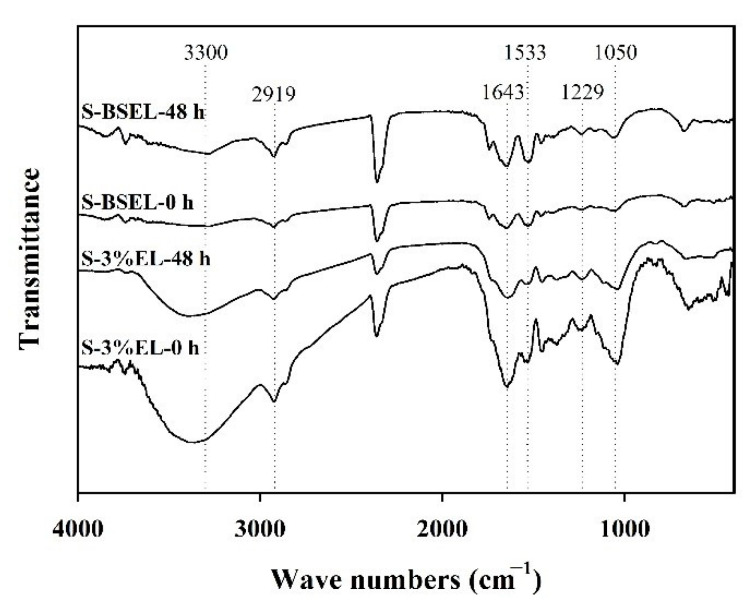
FTIR spectra of the solid-parts separated from the sterilized fermentation medium after inoculation of *L. plantarum* for the four samples: (1) solids of 3% EL in MRS at 0 h (S-3%EL-0 h), (2) solids of 3% EL in MRS at 48 h of fermentation (S-3%EL-48 h), (3) solids of 3% EL in black soymilk at 0 h (S-BSEL-0 h), and (4) solids of 3% EL in black soymilk at 48 h of fermentation (S-BSEL-48 h).

The changes of functional groups of the solids separated from sterilized medium after inoculation of *L. plantarum* by the fermentation were verified by using FTIR analysis for 0 and 48 h of fermentation. The results are shown in Figure 6 for the samples of S-3%EL-0h, S-3%EL-48h, S-BSEL-0h, and S-BSEL-48h. The FTIR spectra of the four samples at bands of 3300, 2919, 1643, 1533, 1229, and 1050 cm^−1^ were compared.

The bands around 3300 cm^−1^ were assigned to O-H stretching vibrations for polysaccharides and phenolic compounds [34], and the order of intensity at 3300 cm^−1^ from strongest to weakest was S-3%EL-0 h > S-3%EL-48 h > S-BSEL-48 h ≈ S-BSEL-0 h, showing more exposure of polysaccharides or phenolic components in the solids after fermentation for the medium 3% EL. With weak intensities at bands around 3300 cm^−1^ for samples S-BSEL-48 h and S-BSEL-0 h, the level of exposure of polysaccharides or phenolic components for the two samples was similar. The band 2919 cm^−1^ was assigned to the C-H stretching vibrations [35], and the intensity of S-BSEL-0 h was the weakest among the four samples, implying the less exposure of aliphalic chains related components in the solid by using more viscous black soymilk.

The band 1643 cm^−1^ was assigned to C=O stretching vibrations [36]. The intensity at band 1643 cm^−1^ for S-3%EL-48 h was weaker than that for S-3%EL-0 h, implying that the exposure of amide I related components in the solid decreased. By using BSEL, the intensity at band 1643 cm^−1^ for S-BSEL-48 h was slightly stronger than that for S-BSEL-0 h, exhibiting that little amide I related components could be transported into the solids during the fermentation.

The band 1533 cm^−1^ was assigned to C-N stretching and N-H bending as the characteristic peak of amide II [36]. The trend at band 1533 cm^−1^ for the four samples (S-3%EL-0 h, S-3%EL-48 h, S-BSEL-0 h, and S-BSEL-48 h) was similar to that at band 1643 cm^−1^, showing the less exposure of amide II for S-3%EL-48 h and S-BSEL-0 h. The weak band 1229 cm^−1^ was assigned to amide III [37], and the trends of intensities for the four samples were also similar to that at bands 1643 cm^−1^ and 1533 cm^−1^. The bands around 1050 cm^−1^ were assigned to skeletal vibrations involving C-O stretching for saccharide [35]. Similarly, the intensity of the band 1050 cm^−1^ for S-3%EL-0 h was stronger than that for S-3%EL-48 h, and the intensity for S-BSEL-48 h was slightly stronger than that for S-BSEL-0 h, displaying a larger exposure of saccharide for S-3%EL-0 h and S-BSEL-48 h. Hence, the changes of functional groups in the solids (mainly the EL) separated from fermented medium by fermentation was verified.

## 4. Conclusions

In this study, a fermented product extract with bioactive compounds and antioxidant capacity from the semi-solid-state fermentation of *E. serratus* leaves and black soymilk by *L. plantarum* BCRC 10357 was explored. The response of *L. plantarim* to the plant niches of *E. serratus* leaves, which contained flavonoid compounds [5], showed the positive adaptation of *L. plantarum* to prolong the stationary growth phase and the stability period of the β-glucosidase activity. For the incubation of *L. plantarum* using black soymilk with *E. serratus* leaves as the medium, the β-glucosidase activity of *L. plantarum* was further enhanced by adaptive regulation. The content of myricitrin, total flavonoid content, and antioxidant activity as ferric reducing antioxidant power in the fermented product extract using black soymilk with *E. serratus* leaves were all higher than that using *E. serratus* leaves alone. This study developed the potential fermented product of black soymilk using *E. serratus* leaves as a nutritional supplement with probiotics.

## Figures and Tables

**Table 1:** Comparison of viable cell counts and β-glucosidase activity of *L. plantarum* BCRC 10357 for 3% EL in MRS broth (3% EL) and 3% EL in black soymilk (BSEL) during 48 h of fermentation ^1^.

Fermentation Medium	Fermentation Time (h)	Viable Cell Counts (log CFU/mL)	β-Glucosidase Activity (U/mL)
3% EL	0	7.57 ± 0.08 ^b^	0.00 ± 0.00 ^b^
3% EL	24	9.63 ± 0.03 ^a^	14.16 ± 1.35 ^a^
3% EL	48	7.53 ± 0.39 ^b^	0.00 ± 0.00 ^b^
BSEL	0	7.73 ± 0.05 ^b^	0.00 ± 0.00 ^c^
BSEL	24	9.48 ± 0.07 ^a^	23.78 ± 1.34 ^a^
BSEL	48	8.02 ± 0.54 ^b^	3.58 ± 1.30 ^b^

^1^ The data were expressed as mean ± standard deviations (*n* = 3). Different superscript letters for the same medium indicate significant differences among the various fermentation times (*p* < 0.05) by Duncan’s multiple range test.

**Table 2:** Effect of *Elaeocarpus serratus* L. leaves on the biotransformation of isoflavones with *L. plantarum* BCRC 10357 ^1^.

Isoflavones (μg/g-FPE)	Fermentation Time (h)		
	0	24	48
Daidzin	1033.01 ± 160.26 ^a,b^	1372.80 ± 271.20 ^a^	763.57 ± 181.47 ^b^
Genistin	853.17 ± 250.65 ^b^	1487.71 ± 290.96 ^a^	1115.25 ± 224.14 ^a,b^
Daidzein	187.78 ± 14.16 ^b^	283.82 ± 49.28 ^a^	339.89 ± 50.17 ^a^
Genistein	54.95 ± 22.99 ^b^	89.55 ± 22.21 ^a,b^	134.58 ± 42.20 ^a^
AI/TI (%)	11.40	11.55	20.16

^1^ The data were expressed as mean ± standard deviations (*n* = 3). Different superscript letters at the same rows were significantly different (*p* < 0.05) by Duncan’s multiple range test. AI (aglycones isoflavones) = daidzein + genistein, TI (total isoflavones) = daidzin + genistin + daidzein + genistein, AI/TI (%) = AI/TI × 100%.

## Data Availability

Data are contained within the article or Supplementary Materials.

## Supplementary Materials

The following are available online at https://www.mdpi.com/article/10.3390/foods10040704/s1, Figure S1: The scheme summarizing the experimental steps, methods, and analysis of this study, Table S1: Comparison of viable cell counts of L. plantarum BCRC 10357 for various %EL in MRS during 48 h of fermentation, Table S2: Comparison of β-glucosidase activity of L. plantarum BCRC 10357 for various %EL in MRS during 48 h of fermentation, Table S3: Comparison of total flavonoids content (TFC) and ferric reducing antioxidant power (FRAP) in fermented product (FP) for various %EL in MRS during 48 h of fermentation by L. plantarum BCRC 10357, Table S4: Comparison of myricitrin content in fermented product (FP) for various %EL in MRS during 48 h of fermentation by L. plantarum BCRC 10357, Table S5: Comparison of total flavonoids content (TFC), myricitrin, and ferric reducing antioxidant power (FRAP) in fermented product extract (FPE) for 3%EL in MRS and 3%EL in black soymilk (BSEL) during 48 h of fermentation by *L. plantarum* BCRC 10357.
