# Supplementary material for: Evaluation of Semi-Solid-State Fermentation of Elaeocarpus serratus L. Leaves and Black Soymilk by Lactobacillus plantarum on Bioactive Compounds and Antioxidant Capacity
Source: Foods. 2021 Mar 26;10(4):704. doi: 10.3390/foods10040704 (PMC8065616; doi:10.3390/foods10040704)
Supplement: Supplementary file 1 [file foods-10-00704-s001.pdf]

Supplementary Materials

Evaluation of Semi-Solid-State Fermentation of *Elaeocarpus serratus* L. Leaves and Black Soymilk by *Lactobacillus plantarum* on Bioactive Compounds and Antioxidant Capacity

Chia-Yu Tsui and Chun-Yao Yang *

Department of Food Science, Fu Jen Catholic University,

No. 510, Zhongzheng Rd., Xinzhuang Dist., New Taipei City 242062, Taiwan (R.O.C.)

*Corresponding author. Tel.: +886 2 29053624; fax: +886 2 22093271.

E-mail address：133810@mail.fju.edu.tw (C.Y. Yang).

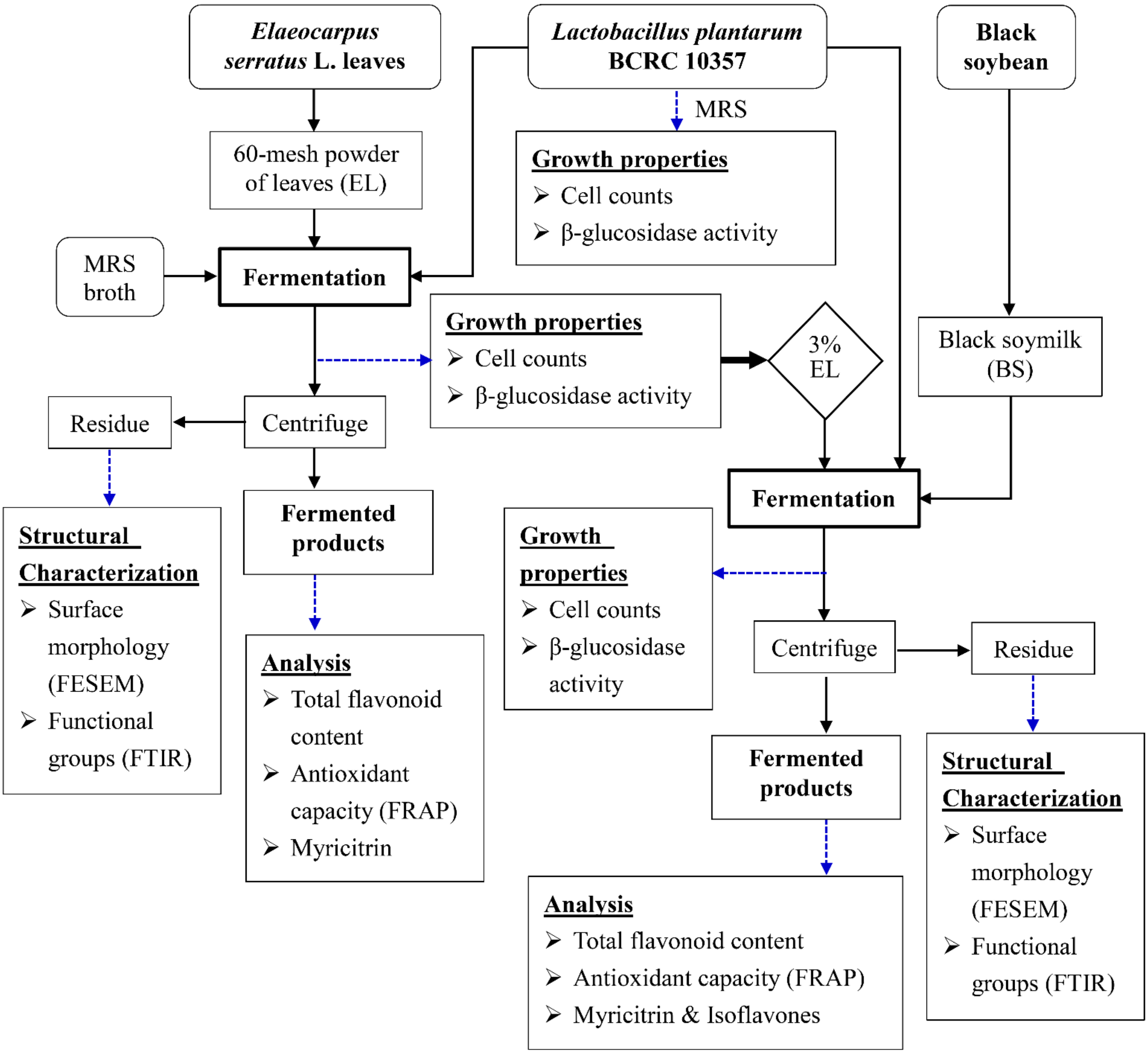

Figure S1. The scheme summarizing the experimental steps, methods, and analysis of this study.

Table S1. Comparison of viable cell counts of *L. plantarum* BCRC 10357 for various %EL in MRS during 48 h of fermentation [1].

**Fermentation time (h)**	**Viable cell counts (log CFU/ml)**				
	**Control**	**0.5% EL**	**1% EL**	**3% EL**	**5% EL**
0	7.69 ± 0.01 ^{Ca}	7.59 ± 0.04 ^{Cab}	7.62 ± 0.07 ^{Cab}	7.57 ± 0.08 ^{Bb}	7.65 ± 0.04 ^{Cab}
12	9.58 ± 0.02 ^{Aab}	9.57 ± 0.10 ^{Aab}	9.66 ± 0.02 ^{Aa}	9.51 ± 0.05 ^{Ab}	9.39 ± 0.02 ^{Bc}
24	9.64 ± 0.04 ^{Aab}	9.73 ± 0.10 ^{Aa}	9.72 ± 0.07 ^{Aa}	9.63 ± 0.03 ^{Aab}	9.55 ± 0.04 ^{Ab}
36	8.47 ± 0.08 ^{Bbc}	8.29 ± 0.62 ^{Bc}	9.05 ± 0.52 ^{Bab}	9.43 ± 0.33 ^{Aa}	9.56 ± 0.04 ^{Aa}
48	7.78 ± 0.10 ^{Cb}	7.43 ± 0.22 ^{Cb}	7.40 ± 0.33 ^{Cb}	7.53 ± 0.39 ^{Bb}	9.52 ± 0.03 ^{Aa}

[1] The data were expressed as mean ± standard deviations (n = 3). Different superscript uppercase letters at the same columns were significantly different ($p < 0.05$) by Duncan's multiple range test. Different superscript lower-case letters at the same row were significantly different ($p < 0.05$) by Duncan's multiple range test.

Table S2. Comparison of β-glucosidase activity of *L. plantarum* BCRC 10357 for various %EL in MRS during 48 h of fermentation [1].

**Fermentation time (h)**	**β-Glucosidase activity (U/ml)**				
	**Control**	**0.5% EL**	**1% EL**	**3% EL**	**5% EL**
0	0.00 ± 0.00 ^{Da}	0.00 ± 0.00 ^{Ca}	0.00 ± 0.00 ^{Ca}	0.00 ± 0.00 ^{Ba}	0.00 ± 0.00 ^{Da}
12	16.56 ± 1.02 ^{Aa}	12.56 ± 1.69 ^{Bbc}	9.21 ± 0.36 ^{Bd}	12.94 ± 0.69 ^{Ab}	10.45 ± 1.51 ^{Bcd}
24	15.47 ± 0.13 ^{Bab}	14.61 ± 1.72 ^{Ab}	18.36 ± 2.38 ^{Aa}	14.16 ± 1.35 ^{Ab}	14.48 ± 2.20 ^{Ab}
36	2.33 ± 0.15 ^{Cc}	0.00 ± 0.00 ^{Cd}	0.00 ± 0.00 ^{Cd}	13.71 ± 0.94 ^{Aa}	7.32 ± 1.07 ^{Cb}
48	0.05 ± 0.09 ^{Db}	0.00 ± 0.00 ^{Cb}	0.00 ± 0.00 ^{Cb}	0.00 ± 0.00 ^{Bb}	5.89 ± 2.06 ^{Ca}

[1] The data were expressed as mean ± standard deviations (n = 3). Different superscript uppercase letters at the same columns were significantly different ($p < 0.05$) by Duncan's multiple range test. Different superscript lower-case letters at the same row were significantly different ($p < 0.05$) by Duncan's multiple range test.

Table S3. Comparison of total flavonoids content (TFC) and ferric reducing antioxidant power (FRAP) in fermented product (FP) for various %EL in MRS during 48 h of fermentation by *L. plantarum* BCRC 10357 [1].

Item	Fermentation time (h)	Ratio of EL			
		**0.5% EL**	**1% EL**	**3% EL**	**5% EL**
**Bioactive Compounds** TFC (μg-RE/g-FP)	0	207.10 ± 56.84 [b]	225.52 ± 76.63 [b]	246.39 ± 38.11 [b]	437.38 ± 54.87 [c]
	24	1215.60 ± 208.62 [a]	1205.06 ± 195.24 [a]	2257.94 ± 472.77 [a]	1682.26 ± 296.09 [b]
	48	1030.28 ± 26.13 [a]	1167.36 ± 104.28 [a]	1865.31 ± 155.87 [a]	2533.41 ± 208.85 [a]
**Antioxidant Capacity** FRAP (mg-TRE/g-FP)	0	1.76 ± 0.31 [b]	1.78 ± 0.45 [b]	1.69 ± 0.36 [b]	2.00 ± 0.09 [b]
	24	6.38 ± 0.41 [a]	8.30 ± 0.56 [a]	9.61 ± 1.37 [a]	7.33 ± 1.51 [a]
	48	6.91 ± 0.96 [a]	7.70 ± 0.83 [a]	8.43 ± 0.87 [a]	9.11 ± 0.80 [a]

[1] The data were expressed as mean ± standard deviations ($n = 3$). Different superscript letters at the same columns were significantly different ($p < 0.05$) by Duncan's multiple range test.

Table S4. Comparison of myricitrin content in fermented product (FP) for various %EL in MRS during 48 h of fermentation by *L. plantarum* BCRC 10357 [1].

Bioactive Compounds	Fermentation time (h)	Ratio of EL			
		**0.5% EL**	**1% EL**	**3% EL**	**5% EL**
Myricitrin (μg/g-FP)	0	34.38 ± 12.07 [c]	49.96 ± 12.28 [c]	115.03 ± 20.79 [b]	159.61 ± 13.53 [a]
	24	232.22 ± 48.65 [c]	339.53 ± 51.83 [c]	1126.70 ± 237.46 [a]	697.96 ± 127.82 [b]
	48	222.16 ±18.35 [c]	317.58 ± 47.09 [c]	1002.41 ± 197.68 [b]	1425.33 ± 108.67 [a]

[1] The data were expressed as mean ± standard deviations ($n = 3$). Different superscript letters at the same rows were significantly different ($p < 0.05$) by Duncan's multiple range test.

Table S5. Comparison of total flavonoids content (TFC), myricitrin and ferric reducing antioxidant power (FRAP) in fermented product extract (FPE) for 3%EL in MRS and 3%EL in black soymilk (BSEL) during 48 h of fermentation by *L. plantarum* BCRC 10357 [1].

Fermentation medium	Fermentation time (h)	TFC (μg-RE/g-FPE)	Myricitrin (μg/g-FPE)	FRAP (mg-TRE/g-FPE)
3% EL	0	2630.42 ± 708.80 [b]	1223.85 ± 325.82 [b]	17.89 ± 4.74 [a]
	24	4052.12 ± 254.76 [a]	2022.36 ± 220.53 [ab]	17.42 ± 1.91 [a]
	48	4106.67 ± 640.33 [a]	2222.91 ± 623.49 [a]	18.59 ± 3.40 [a]
BSEL	0	8670.89 ± 1786.51 [a]	1426.61 ± 119.96 [b]	27.18 ± 2.39 [a]
	24	9267.88 ± 1964.09 [a]	2817.17 ± 435.51 [a]	27.83 ± 3.01 [a]
	48	9643.64 ± 1120.21 [a]	2297.06 ± 328.35 [a]	26.98 ± 2.21 [a]

[1] The data were expressed as mean ± standard deviations ($n = 3$). Different superscript letters at the same columns were significantly different ($p < 0.05$) by Duncan's multiple range test.
